# The regulatory properties of anger under different goal orientations: the effects of normative and outcome goals

**DOI:** 10.1186/s40359-022-00815-7

**Published:** 2022-04-22

**Authors:** Faye Antoniou, Ghadah AlKhadim, Dimitrios Stamovlasis, Aikaterini Vasiou

**Affiliations:** 1 Department of Educational Sciences, National and Kapodistrian University of Athens, Athens, Greece; 2 Department of Psychology, College of Arts, Taif University, P.O. Box 11099, Taif, 21944 Saudi Arabia; 3 School of Philosophy and Education, Aristotle University of Thessaloniki, Thessaloniki, Greece; 4 Department of Primary Education, University of Western Macedonia, Kozani, Greece

**Keywords:** Normative, Outcome goal orientations, Arousal, Anger, Tower of Hanoi, Self-regulation

## Abstract

**Background:**

The present study aimed to evaluate the self-regulatory properties of anger on the performance of individuals under various motivational dispositions using an experimental design.

**Methods:**

The participants were 99 university students who participated in response to extra credit. The performance of the participants was evaluated using the Tower of Hanoi task. Their anger was measured using a facial expression recognition system and arousal was assessed using a heart-rate monitoring device. Two motivational dispositions were assessed: performance goals with normative evaluative standards and performance goals with a focus on outcomes.

**Results:**

The results indicated that a nonlinear function explained the relationship between anger, arousal, and achievement under different goal conditions. Specifically, the Cusp Catastrophe Model showed that anger levels beyond a critical point were associated with the unpredictability of performance during the normative goal condition, suggesting that anger disturbed the relationship between arousal and achievement. Interestingly, a linear model was relevant for explaining the same relationships during the outcome goal condition.

**Conclusion:**

Thus, this study concluded that anger plays a more salient role when coupled with the pressures arising from employing interpersonal evaluative standards.

## Background

Research on motivation and self-regulation [1, 2] has suggested that helping individuals acquire self-regulatory skills promotes adaptability and coping with social, environmental, and academic demands. Zimmerman further proposed that highly motivated individuals can self-monitor their own goals, engage in self-control, and are more academically successful. Successful self-regulation requires remaining focused on a task, thus, having the capacity to regulate both attentional and emotional processes at hand when facing competing alternatives to temporarily distant but important goals. To be successful over the long term, students need to make appropriate choices, engage in goal-directed actions, be efficacious, have self-control and grit, be proactive, and be able to delay gratification. Ample studies confirmed the theses of Zimmerman's theory [3–6]. Among predictors of effective self-regulation, motivation has undoubtedly been a major factor. Below is a description of achievement goal theory and its role in effectively regulating debilitating emotions such as anger. Initially, there is a description of advances in goal theory, followed by a conceptualization of anger as a discrete emotion.

### Achievement goal theory: description and empirical findings

Achievement goal theory originated from the dichotomization of intrinsic and extrinsic motivation as means of elaborating on these two main constructs. At first, two sets of goals were put forth: mastery and performance. Mastery goals place their emphasis and focus on the ongoing mastery of an activity, drawing motivation and positive value from the activity itself. Given that they tap internal processes that reside on value and interest, mastery goals are expected to foster positive emotions and have negative associations with negative emotions. Performance goals, on the other hand, have traditionally focused on proving performance using interpersonal standards of success, thus, mainly by outperforming others. Grant and Dweck, in their study, that distinguished normative and outcome performance-approach goals [7], pointed to the need to distinguish between various types of performance goals through evaluating the motivational focus especially those with and without normative evaluative criteria. They justified this proposition on the fact that normative comparisons are grounded in educational practices using grades, postings, etc. Furthermore, Grant and Dweck found that both types of goals manifested themselves with a less adaptive pattern of cognitive, affective, and behavioral variables than the one manifested by mastery approach goals, and a more adaptive pattern than that manifested by ability validation goals, which, by definition, involve self-worth concerns. Although they reported little effects of normative goals across a host of affective, cognitive, and behavioral processes, they did manifest a positive association with perceived ability, and a negative association with deep processing of the material. Outcome goals were associated with a host of variables, but the pattern of results was mixed including loss of motivation but also active help-seeking.

These findings highlight the need for further investigation of the psychological experiences associated with pursuing normative and outcome performance goals with the main hypothesis put forth suggesting the presence of saliently different emotional experiences. Although both goals focus on performance, the emphasis on absolute performance standards in outcome performance goals compared to the interpersonal social-comparative standards in normative performance goals is likely to guide students in setting different objectives, monitoring and attending on different cues, interpreting feedback in diverse ways, and coping differently when challenged, given the moderating roles of emotions.

### Discrete emotions and anger

Following the distinction between state and trait emotions [8–10], students’ achievement emotions can be seen as affective states that are experienced within specific situations, are brief, are habitual and define students’ current and future emotional tendencies [11, 12]. Studies have found that highly prevalent in classroom settings are the emotions of enjoyment, anger, boredom, and anxiety [13–17]. There is high consensus among emotion theorists that anger is generated by judgments of personal responsibility, especially when the cause of failure is rooted in the lack of effort [18, 19]. Anger is experienced when a learning activity is negatively or positively valued [11] and is often considered to be approach-related, promoting an effort to restore desired states [20–23].

### Anger and self-regulation

Based on evolution theory, anger represented an approach-mechanism to help humans survive through energizing necessary cognitive and physical resources by confronting human or animal threats [24, 25]. Thus, a person may utilize anger by intentionally elevating physiological arousal in order to cope with a threat [26–34] and respond adaptively to environmental pressures [21–23]. Using the emotion as social information (EASI) theory [35], anger results in the provision or relevant and useful information in order to cope with environmental demands in non-academic [36, 37] and academic environments [11, 16, 17, 38]. Therefore, experiencing anger can motivate behavior by representing an approach coping mechanism [39–41]. When anger, however, signals a person’s lack of control to cope with environmental demands [38, 42–44] self-worth threats may become prevalent [45]. Given that performance goals have been linked to self-worth threats, and experiences of hopelessness, incompetence, desperation, and lack of control [46–51] it will be important to evaluate the role of anger when performance goals are operative.

### The nonlinear framework and the regulatory properties of anger

The research work that has established associations among achievement goal theory, emotion, and self-regulatory processes was based predominantly on linear methodologies. There has been evidence that under certain circumstances, specific nonlinear processes are operative, which cannot be captured by the use of linear models. These concern sudden shifts and discontinuities in behavior [52], which can be modeled by fostering a *complex dynamical systems* (CDS) perspective. Part of this nonlinear framework is *catastrophe theory* (CT), a mathematical premise [53] that describes discontinuous changes in a system between qualitatively different states or behavioral modes. These distinct states are the *attractors* of the systems, and represent the behavioral space in which the system moves. Transitions between attractors are nonlinear phenomena that CT models can capture and interpret. The cusp catastrophe model describes shifts between two attractors [54], which, for a cognitive system, might represent optimal versus suboptimal performance. The cusp model posits that changes between two behavioral attractors are predicted by two control parameters: asymmetry (a) and bifurcation (b). The mathematical expression of the cusp is given by the following Eq. (1), which involves a potential function *f*(y; a, b):

1 $$f\left( {{\text{y}};{\text{ a}},{\text{ b}}} \right) \, = {\mathbf{a}}{\text{y}} + {1}/{2}{\mathbf{b}}{\text{y}}^{{2}} - {1}/{\text{4y}}^{{4}}$$

Equation 1 represents a dynamical system seeking optimization expressed by the first derivative (Eq. 2) setting equal to zero:

2 $$\delta f\left( y \right)/\delta y = \, - y^{{3}} + {\text{by }} + {\mathbf{a}}$$

**Fig. 1:**
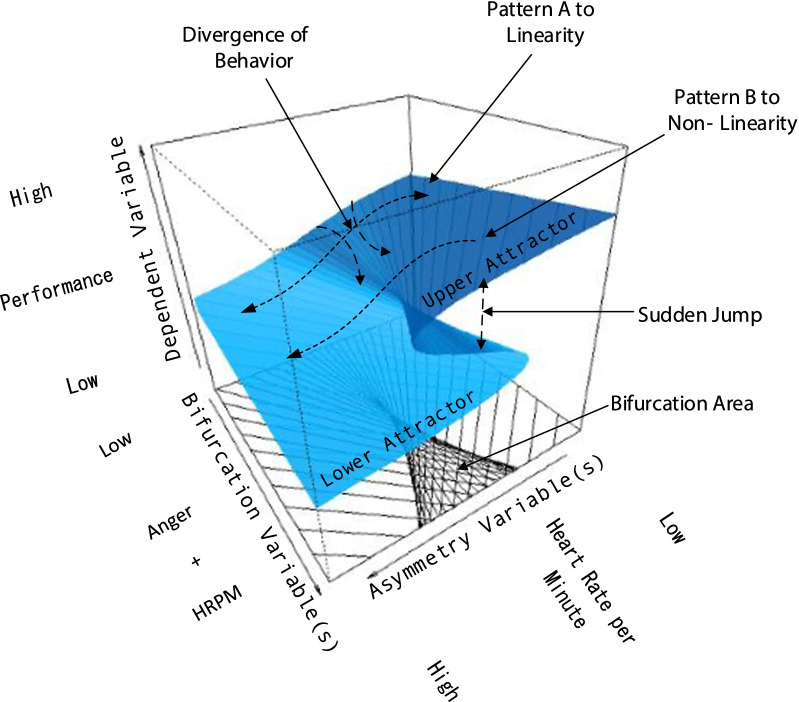
Description of the cusp model within the context of the present study. When asymmetry and bifurcation levels are low, the relationship between the focal variables is expected to be linear (Pattern A). When levels in the bifurcation variable increase beyond a specific critical threshold, Pattern B is expected to be associated with non-linearity. *HRPM* heart rate per minute

The three-dimensional equilibrium response surface of the cusp model derived from Eq. 2, depicted in Fig. 1, describes the patterns of behavior as a function of **a** and **b**. At the back part of the surface, where bifurcation has low values, the behavioral change is smooth and gradual since linear relationships are held between predictors and the outcome. However, when bifurcation increases beyond a critical value, the behavioral variable becomes bimodal, and the changed behavior involves abrupt moves between opposing behavioral modes. The nonlinear effects posited by the cusp model described here are expected to be present during the normative goal condition for which self-regulation failure is imminent given enhanced arousal.

In the present context, in the shift between the attractor of high performance and the attractor of low/sub-optimal performance, under certain conditions, anger can act as a negative emotionality manifestation, and also as a bifurcation variable. Previous research reports have established that during the normative goal condition, which is susceptible to affective influences [55], it is expected that students' self-regulation processes would be disrupted. Furthermore, affective experiences can moderate cognitive performance, as suggested by several theoretical frameworks [56–59]. Thus, cusp modeling is a potential candidate for modeling these behavioral modes.

Owing to the dynamical nature of the self-regulation processes and given the sensitivity of the parameters, extreme values of negative emotion, i.e., anger, may induce nonlinear effects manifested with bimodality, thus introducing uncertainty in the system. Focusing on the role of emotions in situations of achievement, empirical evidence for nonlinear effects has shown that anger, along with other negative emotions, is a potential bifurcation factor as it can drastically affect self-regulation [60]. That study tested the proposition that negative emotions during the pursuit of normative performance-approach goals are associated with uncertainty and chaos in help-seeking behavior through a mechanism of affective dysregulation (AD). Thus, it is reasonable to seek further empirical evidence demonstrating the crucial role of anger; the present study attempts to test this intriguing hypothesis by engaging a novel theoretical framework, that of complex dynamical systems.

### Importance of the present study

Studies have shown that the acquisition of self-regulatory skills plays a salient role in students’ academic endeavors [61–64]. What is less researched, however, is the role of emotions or the interplay between motivations and emotions during task pursuit. As Linnenbrink-Garcia and Pekrun [65] state, there is a need to better understand how emotions unfold and reciprocally relate to motivation, cognitive processes, and academic performance across time.

Anger as a discrete emotion is traditionally perceived as a negative affective state although empirical findings have provided links to also the approach motivational system [66]. Anger has been linked with assertiveness [67, 68], instrumentality [69], and toughness [70, 71]. Similarly, although anger, anxiety, and shame are inherently assumed to reduce intrinsic motivation as negative emotions tend to be incompatible with enjoyment, as implied by interest and intrinsic motivation, they can induce strong motivation to cope with negative outcomes. For example, task-related anger may be assumed to trigger motivation to overcome obstacles [72] in non-achievement situations. Negative activating emotions may impair achievement by reducing the intrinsic value and by producing distracting and irrelevant thinking. However, they may also benefit achievement by strengthening extrinsic motivation. Students’ anger may lead to high levels of subjective control, as well as valuing the need to win [73]. Due to both the engagement of different theoretically based activation schemes and the contradictory empirical findings, it is unclear how anger may be associated with self-regulation of behavior and performance in achievement situations. Thus, the presents study aimed to investigate the self-regulatory properties of anger and physiological arousal as a function of both a challenging task and the induction of various achievement-threatening and non-threatening motivational orientations.

### Hypotheses

This study focused on achievement as a dependent variable assuming that students’ performance is affected by the levels of arousal since a basic stimulation is required to perform a task, as a means of mobilizing the person and orienting him/her toward a goal. Low levels in heart rate per minute (HRPM) likely signal apathy, wherea high HRPM demonstrated enhanced arousal. ModerateHRPM rates (i.e., between 60 and 100 pulses per minute) likely indicate that a person is awakened and mobilizes the cognitive resources necessary to attain self-regulation. Thus, the arousal-based heart rate can serve as the asymmetry factor in a cusp model. However, during the task, the elevated level of arousal can play another role; and given its association with emotion, along with anger, are the variables that have an affective influence and are expected to be potential disruptors of the self-regulation process. Thus, both arousal–heart rate during tasks and anger can jointly act as bifurcation variables. The nonlinear effects sought via the above design are expected to be manifested under only the normative performance-approach goal condition, where changes in negative affectivity during task engagement might lead to the disruption of the self-regulation process. The overall objective of the present study is the comparison of the regulatory properties of normative versus outcome goals predicting performance and positing specific roles for physiological anxiety and anger.

## Method

### Participants and procedures

The participants in the present study were 99 university students (70 females and 29 males) from two state universities. The unequal male to female ratio is due to the overrepresentation of females in education and psychology. They participated in the experiment in exchange for extra credit in their courses in psychology, education, and economics. After being assured about the confidentiality of their responses, participants were provided with a general and least precise purpose of the study as one that intends to evaluate information processing and emotions during achievement situations. Participants were informed that they could withdraw their participation at any time during the experiment, and they were encouraged to not take part in a specific day if they had any health-related concerns (sickness, fatigue, lack of motivation, etc.). The participants answered all questions and signed an informed consent form before commencing the study. First, the participants wore a commercial heart-rate monitoring device and were randomly assigned to one of two experimental conditions. They were subsequently seated individually in front of a computer, and a calibration process ensured proper lighting and the efficacy of the software in capturing their facial expressions accurately. During the calibration process, participants completed a series of self-report scales, read the instructions, and answered task-related questions. Engagement with the task lasted approximately 15 min for the majority of the participants. Participants were then debriefed and thanked for being part of the experiment. There were 50 and 49 participants in the normative goal condition and the outcome goal condition, respectively.

### Measures

#### Achievement task

**Fig. 2:**
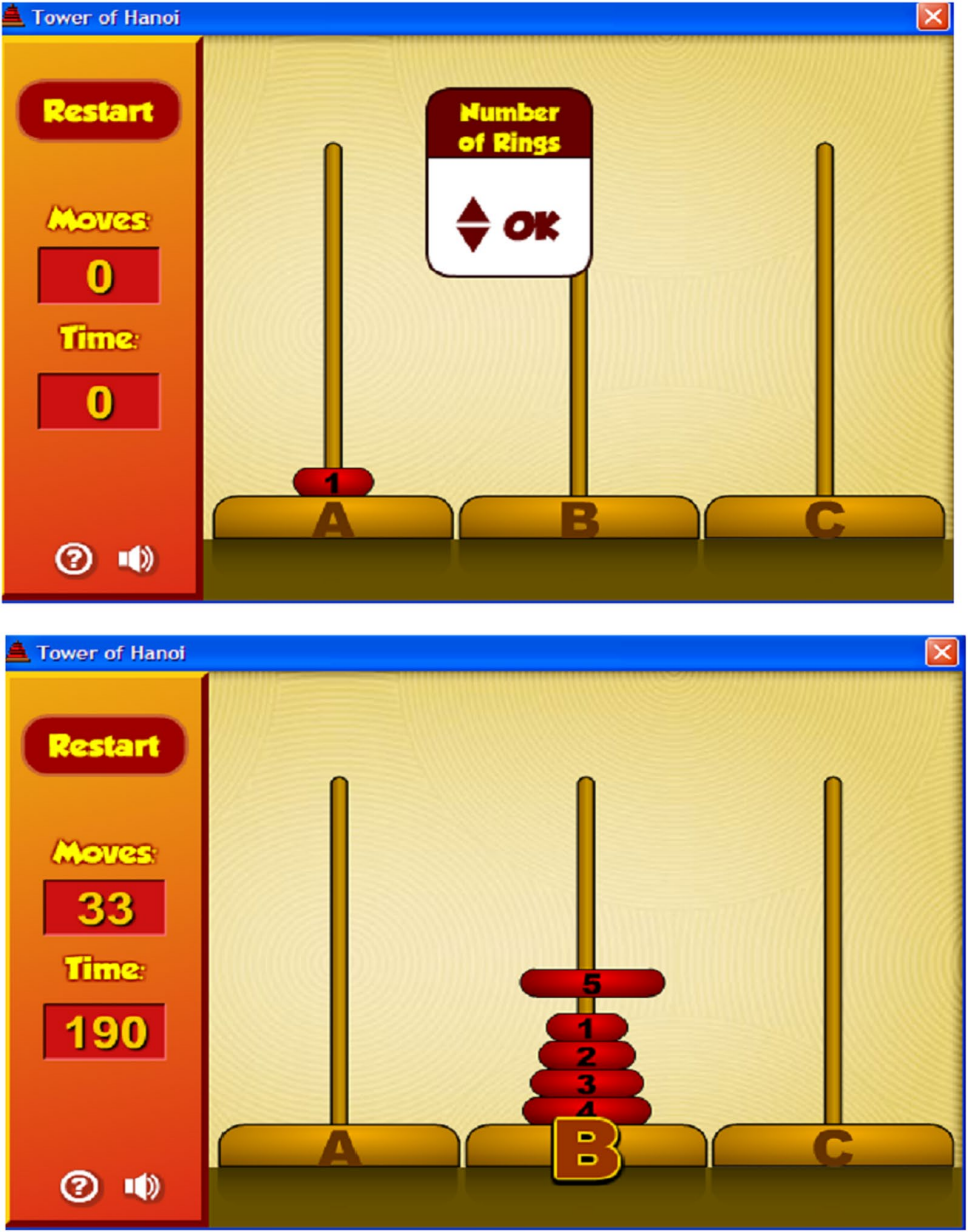
Screenshot of Tower of Hanoi task. Screenshot of the computerized version of the Tower of Hanoi cognitive task (upper panel) and an error in transferring the elements (lower panel)

The performance of the participants in the successful completion of the Tower of Hanoi task was measured. They were required to move the discs from the left to the right using ordered disc sizes (Fig. 2). It was developed in 1883 by Lucas, a French mathematician, as a measure of executive function and information processing capacity [74]; it has also been linked strongly to fluid intelligence [75]. The participant was required to move all discs from towers 1 and 2 onto Tower 3, without, placing a larger disk onto a smaller disk and without moving two discs at the same time. The task commenced when all discs were placed on the left side tower, and only the top disc of the tower could be manipulated at a given time. The task was computerized and the time spent was also monitored. The number of moves was unrestricted, and the participants could make use of help cards that provided cues on how to solve problems. Past research has shown that the Tower of Hanoi task, albeit brief, represents moderate to high levels of challenge in adults (e.g., [76]); thus, it was deemed appropriate to evaluate the emotional manifestation of adaptive motives but more so, of maladaptive motives such as those residing on interpersonal comparisons [77]. The achievement was estimated based on the actual number of towers that the participant could fill up with discs.

### Experimental conditions

The experimental manipulations involved creating conditions related to normative performance goal orientations that are based on interpersonal standards and outcome performance goals that target and value specific outcomes, irrespective of the performance of others [7]. The instructions for goal-framing conditions followed the directions of past research [60, 78–80] and were as follows.

#### Normative performance-approach goals

“This is a challenging task that will take about 15 minutes of your time. The goal is to do better than all other students who take this task. We would like you to try and outperform everyone else. At the end of the experiment, we will post everyone’s scores on the bulletin board, from best to last. So, try to do your best and outperform everyone else.”

#### Outcome performance-approach goals

“This is a challenging task that will take about 15 minutes of your time. The goal is to perform as best as possible on this task. We would like you to try and do your best in this task according to your own standards.^1^”

### Validation of experimental manipulations

At the end of the task, participants had to verbally repeat the instructions posited by the experimenter; that is, they were asked to repeat what they were trying to accomplish. Ninety-eight percent of the participants (n = 97) repeated the instructions properly and comprised the sample of the present study; two were excluded because they failed to recall the initial directions.

#### Arousal

A commercial valid heart-rate monitoring device was used to assess the participants’ physiological responses during the execution of the task. Research has shown that the specific model (Polar 810i) has ample testimonies on its validity [81, 82]. Data were collected at 10-s intervals and were subsequently transferred to the database for analysis. Both the baseline heart rate (BLHR) and the heart rate during the task (HRDT) were recorded and used.

#### Anger

The participants’ facial expressions were assessed using a commercial application, “Face Reader.” The face reader is a system comprising both hardware and software aimed at providing a valid measurement of a person's facial expressions. It utilizes an artificial neural network that aims to detect Ekman’s six basic emotions: happiness, sadness, anger, surprise, fear, and disgust [83–85]. In the present study, only anger was manipulated. Accuracy rates of face recognition have been reported to have a mean of 89% across emotions [86].

The identification of emotion processing involves four steps: (a) the application of a template to face location, (b) the utilization of the active appearance model to reconstruct the face using 55 key elements, (c) estimation of various levels between areas, which is estimated using a large database containing annotated images, and (d) the final classification of facial expressions based on both the training and application of an artificial neural network, which was also initially trained to classify the six basic emotions. The process of recognition considers demographic and facial information such as participant’s age, ethnicity, gender, amount of hair, the existence of beards, and/or the wearing of glasses. All data were collected in real-time and per second, following instrument calibration.

### Data analysis

Data analysis for cusp catastrophe was carried out using the cusp probability density function [87]. This analysis uses the maximum likelihood [88] and is performed with *a cuspfit* in R. The cuspfit utilizes numerical procedures for parameter estimates by minimizing a negative log-likelihood function, and a comparison of the cusp model with the alternative linear and logistic model is provided using AIC (Akaike's Information Criteria) and corrected AIC and BIC (Bayesian Information Criteria) indices [89]. In the present analysis, the achievement was the dependent measure, while arousal–baseline heart rate was the asymmetric variable and arousal–heart rate during the task along with anger were the bifurcation variables. Evidence in favor of the cusp model is indexed by non-normal responses within the bifurcation area, data within the bifurcation area being approximately 10% of the total number of data or more, and the presence of skewed distributions outside the bifurcation area (left and right sides of the response surface). Furthermore, lower information criteria values (i.e., using AIC and BIC) are expected when the cusp model is the preferred model compared to the competing models. Last, differences between nested linear and cusp models using a chi-square difference test are also expected. The model was run using standardized variables for interpretation purposes and separately for normative and outcome performance goal conditions. The results are shown in Table 1, with parameter estimates for intercepts and slopes for both goal conditions. A power analysis was estimated for a predictive model using 3 independent variables. For power levels equal to 80% and a medium effect size (f-squared = 0.15), a sample size of *n* = 77 is required using a two-tailed test with alpha = 5%. Thus, the present study involving a sample size of n = 83 possessed adequate levels of power when testing our multivariate models’ effects.

**Table 1:** Parameter estimates of the cusp model for the prediction of achievement from physiological arousal and feelings of anger in the normative and outcome goal conditions

Variable	*B*	*S.E.*	*Z*-value	*p-value*
*Normative performance goal condition*				
a(Intercept)	− 0.067	0.274	− 0.248	0.804
a(Baseline Heart Rate)	0.267	0.170	1.575	0.115
b(Intercept)	0.459	0.006	81.625	0.001***
b(Anger)	0.521	0.035	15.091	0.001***
b(Heart Rate During Task)	0063	0.032	1.970	0.049*
w(Intercept)	− 0.027	0.168	− 0.160	0.873
w(Achievement)	0.915	0.066	13.838	0.001***
*Outcome performance goal condition*				
a(Intercept)	0.343	0.328	1.046	0.296
a(Baseline Heart Rate)	0.149	0.165	0.901	0.368
b(Intercept)	− 0.321	0.016	− 19.489	0.001***
b(Anger)	0.119	0.201	0.590	0.555
b(Heart Rate During Task)	0.401	0.265	1.511	0.131
w(Intercept)	0.304	0.168	1.810	0.070
w(Achievement)	0.787	0.050	15.810	0.001***

***p < . 001; **p < .01; *p < .05, two-tailed tests

## Results

### Goals, physiological arousal, anger and achievement

**Fig. 3:**
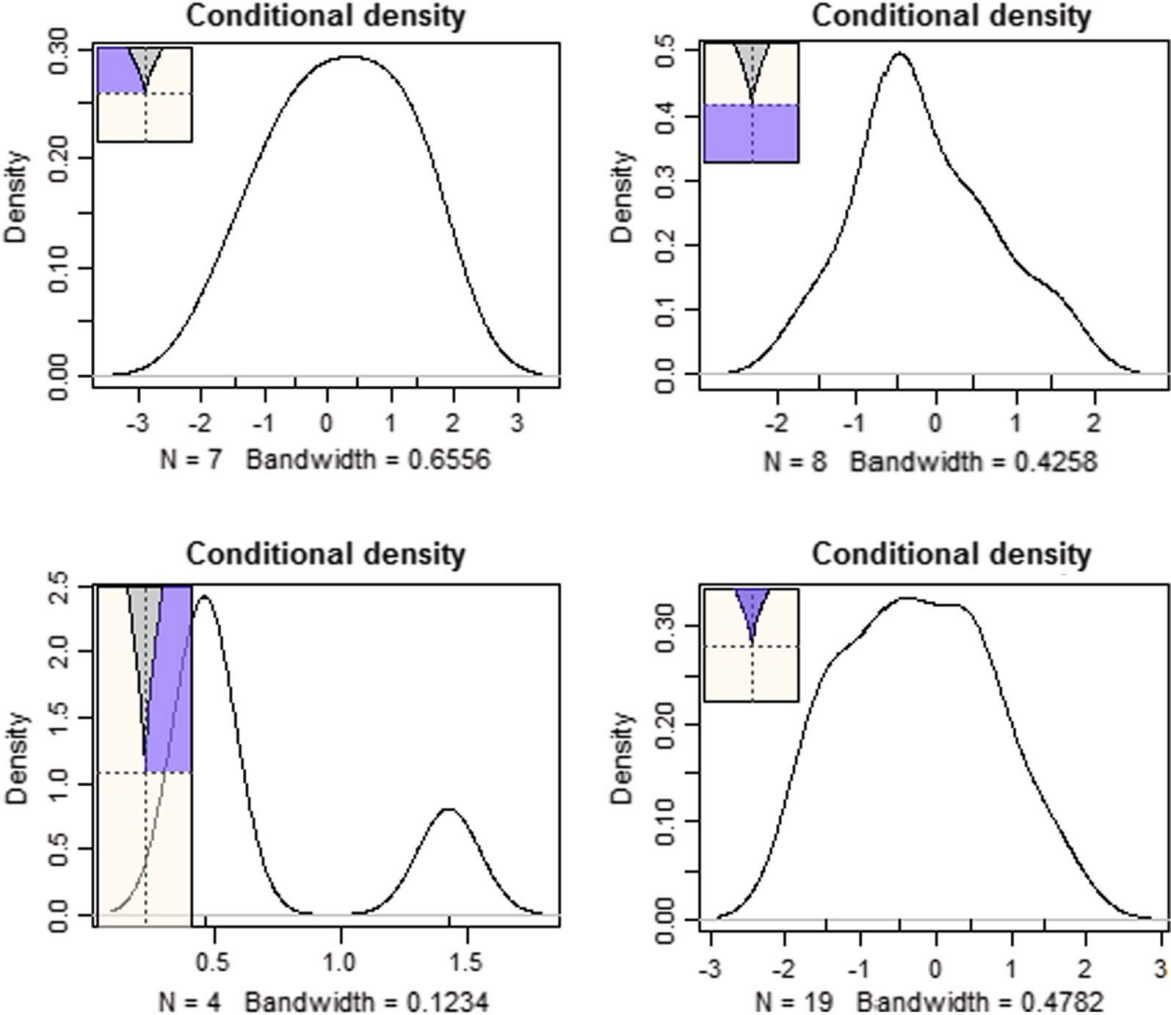
Conditional densities of observations at various locations on the space surface. The presence of multimodality and skew are evident at various locations in the response surface as they are expected when the cusp model fits the data well. The area in which non-normality in the form of spikes is expected is within the bifurcation area (bottom right), which is also difficult to attain when the expectation is that only 10% of the observations are required to fall in this area. Nevertheless, the shape of the function suggests the presence of almost a uniform distribution, which again deviated considerably from the normal curve that is expected when linear relationships are evident

**Fig. 4:**
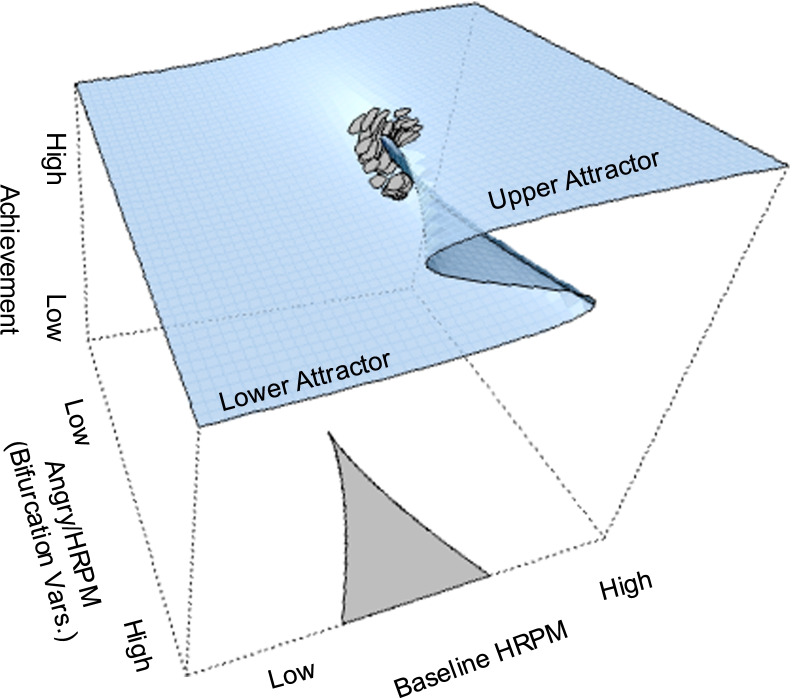
Cusp catastrophe model with observations moving from the stable upper attractor to the lower attractor thus, entering the bifurcation area of uncertainty and unpredictability. Thus, the fold of the upper surface indicates that observations fall within the uncertainty and unpredictability area

As shown in Table 1, in the normative performance goal condition, both bifurcation parameters, heart rate per minute during the task, and observed manifestations of anger were significant determinants of a cusp (*b*_HRPM_ = 0.063, *p* < 0.05; *b*_Anger_ = 0.521, *p* < 0.001). The immediate interpretation from the analysis is that when angry facial expressions are coupled with physiological anxiety, achievement behavior becomes unpredictable and enters a state of uncertainty before eventually again regulating the system toward a stable behavior. Table 2 shows the superiority of the cusp model over the linear and logistic models using the information criteria. Specifically, the cusp model was associated with lower values of the AIC, corrected AIC indices, and BIC. Furthermore, the R-squared values were 33.6%, 15.9%, and 7.7% for the cusp, logistic, and linear models, respectively, indicating the superiority of the cusp model. A Chi-square test indicated the presence of significant differences between the cusp and linear models [*χ*^*2*^(2) = 7.513, *p* < 0.05] and between the logistic and cusp models using a one-tailed test [*χ*^*2*^(1) = 3.340, *p* < 0.05, one-tailed test]., adding further evidence to the preference for the cusp model. Figure 3 shows the density functions in various areas of the control space. As expected, skew and bimodality or multimodality, and generally the absence of normality, were observed across various areas in the response surface [89]. Finally, Fig. 4 shows that most observations lie on the upper and lower surfaces, with some oscillating from the upper to the lower surface and within the bifurcation area.

**Table 2:** Estimates of model fit for linear, logistic, and cusp competing models by goal condition

Model tested	N. of Par	AIC	AIC_c_	BIC	R^2^ (%)
*Normative performance goal condition*					
Linear model	5	116.251	118.126	124.439	7.8
Logistic model	6	114.735	117.445	124.561	15.9
Cusp model	7	112.738	116.472	124.201	33.6
*Outcome performance goal condition*					
Linear model	5	231.282	232.282	243.192	3.4
Logistic model	6	225.562	226.712	239.854	12.3
Cusp model	7	233.696	235.252	250.370	− 0.02^†^

N = number of estimated parameters; AIC = Akaike information criterion; AIC_c_ = corrected Akaike criterion; BIC = Bayesian information criterion

^†^As noted in the text R-square values using cusp can take on negative values and that was evident when modeling achievement in the outcome performance goal condition

During the outcome performance goal condition, the cusp was the worst model to fit the data compared to both the linear and logistic models using information criteria (see Table 2). The linear model accounted for 3.4% of the variance, logistic, 12.3%, and cusp, 0%. Furthermore, the difference between linear and cusp models using the chi-square test was found to not be significant (*χ*^*2*^(2) = 1.585, *p* > 0.05), but the difference between cusp and logistic models was significant pointing to the superiority of the latter [*χ*^*2*^(1) = 6.134, *p* < 0.05], suggesting again that the cusp model was not preferred amongst all competing models. When looking at the magnitude of the coefficients, none of the bifurcation variables were linked to achievement in a significant manner within the cusp model. Therefore, the results supported the research hypothesis that the cusp model is most appropriate for normative evaluative standards and the linear model for outcome goals.

## Discussion

The present study aimed to test a nonlinear hypothesis related to the role of anger under achievement pursuits, specifically during two contexts of performance goals: those associated with normative evaluative standards and those associated with outcome evaluative standards. It was shown that the pursuit of normative performance-approach goals is associated with discontinuities in students’ achievement through a mechanism of affective dysregulation.

### Model interpretation and links to theory

Although the linear relationship between achievement and baseline HRPM was not empirically supported, the roles of HRPM during the task and anger as bifurcation variables were demonstrated in this study. At low values of negative affectivity, low arousal, and low anger, changes are smooth, linear, and predictable. When anger covaries with high arousal and both reach a critical threshold value, the system reaches uncertainty, turmoil, and demonstrated sudden shifts between the behavioral attractors. This critical event suggests the presence of a *bifurcation point* which drives behavior to unpredictability as it is being drawn across a wide range of modes (see Fig. 4). In other words, within this area of uncertainty (termed *inaccessibility*) behavior can take on any form, adaptive or maladaptive. In dynamic systems theory, this phenomenon is termed *hysteresis,* as individuals with equal levels in the independent variables (asymmetry and bifurcation) may end up with diverse academic outcomes, before reaching self-organization [90].

The behavior observed post-task, is a result of a complex course of actions involving circular interactions among cognitive and affective components, similar to the self-regulation process described earlier [1, 2, 6]. Interestingly, behavior cannot be explained using a linear understanding of relationships between variables but only using dynamical systems. The nonlinear behavior is explained by the dynamic character of the system and the underlying self-organization mechanics (Molenaar and Raijmakers, 2000; [91]). Self-organization theory portrays the interactions among the contributing components of a system that operates in a state of high entropy and far-from-equilibrium conditions, where it functions more efficiently and adapts to environmental fluctuations (Prigogine and Stengers, 1984; [92]). The present empirical work contributes to theory development, embracing achievement goal theory, and emotions within their theses. In this framework, the regulatory properties of anger were elucidated in goal conjunction with performance orientations/structures. Furthermore, this work demonstrated the existence of links and interactions among cognitive, affective, and attitudinal components in human psychological processes.

From the preceding analysis, it was shown that for performance-oriented students under normative “pressures”, affect may dominate their ability to self-regulate and achieve optimal performance outcomes. This is in line with anecdotal reports on affective dysregulation observed for performance-oriented individuals (e.g., [93]). The other important contribution of this study is the suggestion that concomitant arousal (HRPM) and anger under the normative performance goal condition explain achievement using non-linear terms. This empirical evidence supports the theoretical perspective that emotional dysregulation leads to behavioral dysregulation and subsequent unpredictability in behavior. For low levels of arousal and anger, a linear relationship is anticipated as low levels of arousal that do not become stressful may not be capable of seriously distorting individuals’ performance. When arousal and anger surpass a critical threshold, the self-regulation mechanism is excessively disturbed, so students’ performance becomes unpredictable as volitional behavior is governed by heavy tasks and emotional demands. Unsurprisingly, under outcome goals, levels of arousal and anger seem easily manageable by psychological systems that succeed in maintaining behavior within the linear regime, avoiding unanticipated sudden transitions. Hence, it can be inferred that severe affective experiences under normative pressures manifested with concomitant effects of both physiology and facial reactions are likely to be linked to self-regulation failure.

The present study’s findings deviated markedly from previous reports (e.g., [7]), which suggested the unification of the two types of performance goals. Thus, it can be said conclusively, that two divergent motives focus on performance outcomes; however, a direct link to normative evaluative standards is associated with emotional dysregulation, cognitive overload, and decrements in performance.

Another important inference that can be drawn from the present findings is the peculiar role of anger under different circumstances. For example, anger has been found to trigger aggression [94] and goal-directed behavior [95] but also can help to motivate individuals during task engagement [96, 97]. Thus, anger can act in both approach [98] and avoidance terms [99]. In the current study, anger was linked to self-regulation failure.

The present study has several limitations. First, the present findings need to be replicated with future samples to ensure stability of the results and generality to additional populations. Second, an “angry” facial expression may represent divergent motivational behaviors. For example, a person may react angrily and then focus and persist or be distracted and avoidant. In the present study we did not test for different manifestations of anger. Third, there may be other important factors and their interactions (e.g., past failures, hopelessness, helplessness) that may be responsible for a total “shut down” and failure, which should be investigated along with achievement goals in future studies. Fourth, crucial mechanisms that are responsible for the interdependencies between the two motives need to be explored under additional cooperative and competitive structures, including tests for the mediating roles of effort and persistence (e.g., [100]). Last, the analytical methodology implemented here has also not been free of criticism [101, 102]. We believe that the findings of the present study, however, open up a new avenue of investigations that can explore within the CDS framework, the emotional links to self-regulation processes in academic and social behaviors, in the context of goal orientations described by Grant and Dweck [7] and in the re-conceptualized frameworks of achievement goal theory put forth by Elliot and Murayama [103]. It is concluded that exploring the role of anger and negative affectivity within complex dynamical system theory will shed light on many aspects of human self-regulation mechanisms involving emotional and cognitive processes and outcomes.

## Data Availability

Data are available from the following repository which includes an SPSS data file and a readme file with a description of the relevant variables. It is located here: https://github.com/GS1968/Anger.

## Footnotes

1 Outcome goals have often been defined using absolute standards of success such as good grades or attaining a specific level of performance. In the present study, the absolute standard (i.e., achieve the best possible performance) was supplemented with a perceived emphasis on the judgment of that absolute standard. This extension has been implemented in order to induce a goal condition for which intra-individual rather than inter-individual standards of performance would be tested.
